# Effect of Residual Plastic Strain on the Fatigue Failure Mechanism and Service Life Prediction of Dented X80 Pipelines

**DOI:** 10.3390/ma19050967

**Published:** 2026-03-03

**Authors:** Peng Ren, Yafang Fu, Jifan He, Naixian Li, Li Zhu, Youkai Gu, Youcai Xiang, Bin Jia

**Affiliations:** 1 School of Mechanics and Aerospace Engineering, Southwest Jiaotong University, Chengdu 610031, China; rp386462@163.com; 2 School of Materials Science and Engineering, Southwest Jiaotong University, Chengdu 610031, China; fuyafang@my.swjtu.edu.cn; 3 School of Civil Engineering and Architecture, Southwest University of Science and Technology, Mianyang 621010, China; linaixian23@gscaep.ac.cn (N.L.); zhuli@swust.edu.cn (L.Z.); xkk125670@163.com (Y.G.); xyc113579@126.com (Y.X.)

**Keywords:** X80 pipelines, deformed dent, residual plastic strain, failure mechanism, fatigue life prediction

## Abstract

**Highlights:**

**What are the main findings?**

- Higher pre-strain significantly reduces fatigue life of X80 steel.
- Pre-strain increases local strain concentration at the shoulder region.
- Crack initiation is accelerated under higher Δ*p* loading.

**What are the implications of the main findings?**

- Pre-strain history must be considered in fatigue assessment.
- Residual deformation reduces resistance to cyclic loading.
- Findings support safer pipeline design under internal pressure.

**Abstract:**

In the field of oil and gas transportation, X80 pipelines are susceptible to localized plastic deformation caused by mechanical impact or geological activity. This leads to the formation of dents and the introduction of pre-strain, thereby affecting the structural integrity and fatigue life. This study systematically investigates the influence mechanism of pre-strain on the high-cycle fatigue performance of dented regions in X80 steel. Fatigue tests conducted across pre-strain levels of 1%, 2%, and 3% revealed that the induced plastic strain significantly degrades fatigue performance. Under constant stress amplitude, fatigue life decreases markedly with increasing pre-strain, a trend driven by the accumulation of micro-damage. Furthermore, a parametric *P-S-N* curve model that incorporates both pre-plastic strain and reliability was developed, providing a basis for quantitatively assessing the impact of pre-strain. By combining finite element analysis with the Smith-Watson-Topper (SWT) critical plane method, it was predicted that fatigue cracks in unconstrained dent primarily initiate at the dent periphery, with the critical plane orientation perpendicular to the circumferential direction, which aligns well with field observations. Parametric analysis indicates that the maximum operating pressure is the dominant factor affecting the fatigue life of the dented pipelines. This research elucidates the material-level fatigue failure characteristics of dented X80 pipelines and provides theoretical insights for life prediction and engineering protection.

## 1. Introduction

**Figure 1:**
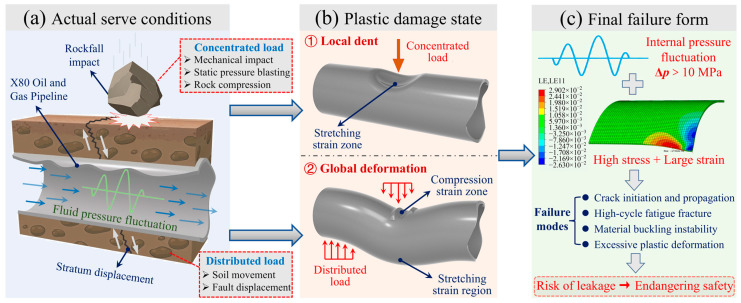
Schematic diagram of plastic damage and fatigue failure of X80 oil and gas pipelines.

X80 pipeline steel, known for its high strength and excellent fracture toughness, is widely used in oil and gas transmission pipelines [1]. These pipelines frequently traverse complex geographical terrains, where they are subjected to various external loads that can induce varying degrees of mechanical damage. Recent studies have also highlighted complex service stress states, such as those caused by in-service welding residual stresses in X80 pipelines, which further complicate the material’s fatigue behavior under operational loads [2]. Among the various forms of mechanical damage, denting is one of the most prevalent forms of such damage, introducing localized stress concentrations and plastic strain in the affected pipeline sections [3,4]. Under cyclic internal pressure, these dented regions experience accelerated fatigue crack initiation, potentially leading to a significant reduction in service life and leakage of the transported medium [5,6], as illustrated in Figure 1. Therefore, investigating the fatigue life of dented pipelines is of considerable practical importance.

Current methodologies for predicting the fatigue life of dented pipelines primarily encompass full-scale dent fatigue tests, finite element analysis (FEA), and semi-empirical formulas, such as those summarized in the Pipeline Defect Assessment Manual (PDAM). The PDAM outlines two commonly used semi-empirical approaches: the EPRG (European Pipeline Research Group) and the SES (Structural Engineering Services) method. Both approaches are based on *S-N* curve fatigue models and employ stress concentration factors to adjust fatigue life predictions for dented pipelines, while also accounting for mean stress effects. Although these methods generally show reasonable agreement with practical engineering data and experimental results [7,8], they often yield conservative estimates for dent fatigue life [9]. Arumugam et al. [10] estimated fatigue crack growth coefficients using full-scale test data and FEA, and proposed a predictive model for the fatigue life of shallow dents with corrosion cracks. Bolton et al. [11] analyzed strain variations from finite element models to predict fatigue crack propagation in dented pipelines. Additionally, Paiva et al. [12,13] demonstrated that the Manson-Coffin fatigue equation can effectively predict the fatigue life of dented pipelines.

Most prior investigations have concentrated on fatigue life prediction based on *S-N* curves combined with stress concentration factors. However, pipelines subjected to internal pressure primarily experience biaxial (axial and circumferential) stress–strain states, which classifies the problem as multiaxial fatigue. The stress conditions in dented regions are particularly complex, making direct fatigue life calculation challenging. The critical plane method offers an effective approach to translating multiaxial fatigue problems into an equivalent uniaxial framework, enabling accurate prediction of fatigue failure locations, modes, and lifetimes. As a result, it has been widely adopted in fatigue analysis. Wang et al. [14] developed a three-dimensional transient finite element model for wheelsets and fixed frogs under various complex loading conditions and applied the critical plane method to predict the fatigue crack initiation life of fixed frog components, successfully forecasting crack location and morphologies. Vantadori et al. [15] utilized the critical plane method to accurately predict the fatigue life and initial crack orientations of 316 and 430 stainless steels under multiaxial loading. Furthermore, Ma et al. [16] improved the critical plane method by refining the damage plane and damage parameters, and validated its accuracy in predicting the fatigue life of notched TC4 and GH4169 specimens. Nevertheless, the application of the critical plane method to the fatigue analysis of dented pipelines remains relatively limited.

The plastic strain induced by denting significantly affects pipeline fatigue life, and the effect of pre-strain on fatigue performance varies among different steel grades. Xu et al. [17] observed that the fatigue life of X80 welded joints slightly increased with pre-strain level under identical cyclic loading, despite a reduction in the crack propagation period. Walker et al. [18] reported that a 10% pre-strain led to a tenfold increase in the fatigue resistance of FB600 steel compared to the substrate, while DP600 steel exhibited a 2.5-fold increase in fatigue life under similar pre-straining conditions. Conversely, experiments by Anandavijayan et al. [19] revealed that the fatigue life of S355 structural steel decreased with increasing pre-strain, although the fatigue crack propagation rate remained relatively constant. Most existing studies have focused primarily on static load-bearing behavior, yet the multiscale relationship between pre-strain effects and structural fatigue under cyclic pressure remains poorly understood. In addition, efficient and high-fidelity approaches for predicting fatigue crack initiation in dented pipelines are still absent. Therefore, investigating the effect of pre-strain on the fatigue life of X80 pipeline steel is crucial for assessing the service life of dented pipelines.

This study investigates the fatigue life of unrestrained dented X80 pipelines via an integrated approach combining experiments, finite element analysis, and the SWT critical plane method. The effects of pipeline thickness, dent depth, and fluctuating internal pressure on the fatigue life were evaluated. The findings provide valuable guidance for damage protection and fatigue life assessment of dented pipelines.

## 2. Materials and Methods

### 2.1. Material and Pre-Strain Treatment

All specimens were machined from an unserved X80 natural gas pipeline with a diameter of *Φ*1219.0 mm and a thickness of 26.4 mm, with their axis aligned parallel to the pipeline’s axial direction. As specified in relevant standards [20,21], specimens with a gauge length of 75 mm and a diameter of 7 mm were prepared (Figure 1a). The chemical composition and mechanical properties of the X80 steel are summarized in Table 1 and Table 2, respectively.

To investigate the effect of pre-strain on fatigue performance, standard tensile specimens were pre-tensioned using a universal testing machine (CMT5150, Meister Industrial System Co., Ltd., Shenzhen, China), as shown in Figure 1b. Based on the tensile stress–strain response of the material, three pre-strain levels were selected: ε_pre_ = 1.0%, 2.0%, and 3.0%. Six specimens were prepared for each group to ensure statistical reliability in subsequent fatigue tests. During pre-straining, an extensometer was attached to the gauge section to monitor the deformation. As shown in Figure 1c, the specimens were loaded under displacement control at a constant rate of 0.5 mm/min until the target displacement (0.2 mm, 0.4 mm, or 0.6 mm) was reached, followed by load-controlled unloading to 100 N. The final plastic strain was determined from the extensometer readings.

### 2.2. Fatigue Test Procedure

Axial fatigue tests were conducted on both as-received and pre-strained specimens in accordance with standard [20], using a low-frequency servo-hydraulic fatigue testing machine (RG-9025, REGER CO., LTD., Shenzhen, China). A total of 24 specimens were divided into four groups: as-received (Original) and pre-strained to 1%, 2%, and 3%. Each group was tested at six distinct stress levels. Given the lack of established data on high pre-strain effects, an initial stress amplitude of 400 MPa was used for all groups and subsequently adjusted based on preliminary results. All tests were conducted under fully reversed loading (stress ratio *R* = −1), with a sinusoidal waveform at a frequency of 10 Hz.

**Figure 2:**
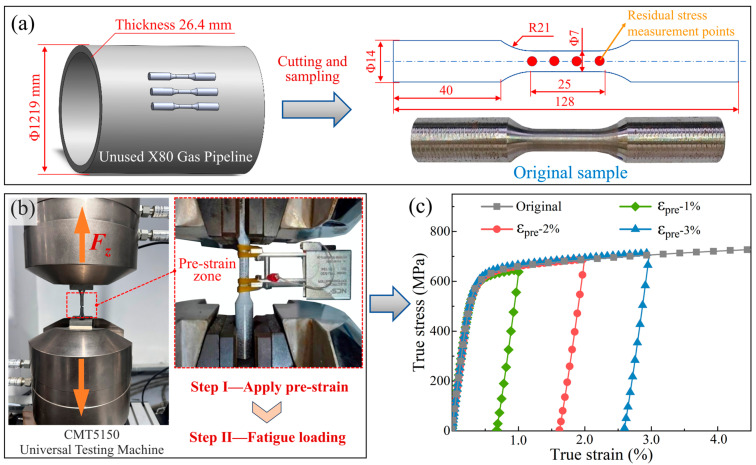
Schematic of X80 pipeline steel specimen extraction and residual stress test points (**a**), photograph of the pre-strain loading setup (**b**), and loading-unloading curves under different strain rates (**c**).

To evaluate the effect of residual stress on fatigue behavior, surface residual stresses were measured before and after fatigue testing using an X-ray diffractometer (Pulstec μ-X360s, Japan) based on the sin^2^ψ method. As shown in Figure 2a, the axial residual stresses were measured at four equally spaced points along the middle section of the specimen surface, with an effective penetration depth of approximately 5–10 μm; the averaged value is reported. Fracture surfaces of specimens tested at a maximum cyclic stress of 400 MPa were examined using scanning electron microscopy (SEM, JMS-6610, JEOL (BEIJING) CO., LTD., Beijing, China) to identify crack initiation sites. For EBSD characterization (C-Nano, Oxford Instruments, Abingdon, UK), the fractured cross-sections were first mechanically polished with a 0.5 μm diamond suspension and then electropolished. EBSD scans were conducted at an accelerating voltage of 30 kV and a magnification of 100×, with a hit rate of 98.17%. A specimen tilt angle of 70° and an acquisition speed of 24.93 Hz were implemented to test the material’s crystallographic features, including crystal structure, grain orientation, grain size, and stress distribution.

### 2.3. Fatigue Data Analysis and S-N Curve Modeling

The *S-N* curve in the medium-to-high cycle fatigue regime is commonly described by a three-parameter power function [23,24]. In this study, the three-parameter power function model incorporating residual strain (Equation (1)) was developed [25]. Fatigue test data for different pre-strain levels were analyzed using the single-point likelihood method, which is a widely accepted statistical approach for constructing probabilistic *S-N* curves from limited datasets. Within this framework, the logarithm of fatigue life is assumed to follow a normal distribution, i.e., fatigue life is lognormally distributed [23,26,27]. The regression parameters and the standard deviation of the logarithmic life are estimated simultaneously from the entire dataset via maximum likelihood optimization. Consistent with conventional probabilistic *S-N* modeling, the standard deviation is assumed to be constant across stress levels (homoscedasticity). Accordingly, the data scatter is not evaluated from repeated tests at individual stress levels, but is inferred from the global statistical fitting of all fatigue data. Based on the estimated distribution parameters, *P-S-N* curves for various reliability levels are constructed by incorporating the corresponding standard normal deviates into the model (Equation (2)), thus generating families of reliability-dependent *S-N* curves.

(1) $${\left(S-S_{0}(\varepsilon_{\mathrm{pre}})\right)}^{m(\varepsilon_{\mathrm{pre}})}N=C(\varepsilon_{\mathrm{pre}})$$

(2) $$N_{p}{\left(S-S_{0}(\varepsilon_{\mathrm{pre}})\right)}^{m(\varepsilon_{\mathrm{pre}})}S^{m^{’}(\varepsilon_{\mathrm{pre}})}=C_{p}^{’}(\varepsilon_{\mathrm{pre}})$$

where *S*_0_, *m*, and *C* are material constants. Since fatigue properties evolve with pre-strain (*ε*_pre_), these constants are modified to be functions of pre-strain level: *S*_0_(*ε*_pre_), *m*(*ε*_pre_), and *C*(*ε*_pre_). Consequently, for a probabilistic analysis, parameters such as *m*_p_^’^(*ε*_pre_) and *C*_p_^’^(*ε*_pre_) are introduced and determined from the data.

The *P-S-N* curves derived for different strain levels serve as a fundamental basis for subsequently developing empirical formulas that describe the relationship between *S-N* curve parameters and pre-plastic strain.

### 2.4. Finite Element Modeling of the Dented Pipeline

**Figure 3:**
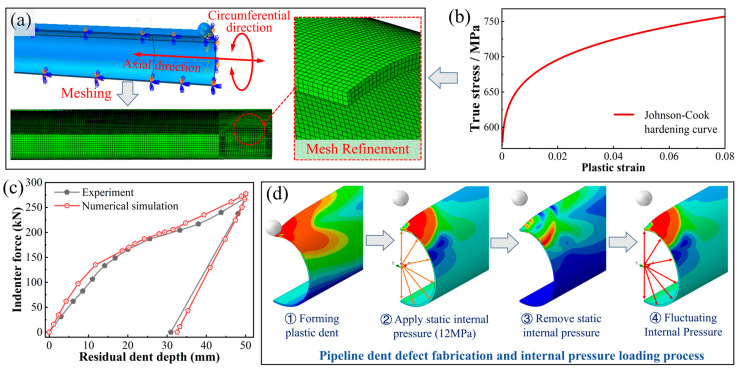
Finite element modeling and simulation process of the pipeline dent: FE model and mesh of the dented pipeline (**a**); Johnson-Cook hardening curve (**b**); Validation against experimental data (**c**); Simulation loading process (**d**).

A finite element model of an unconstrained dented pipeline was established in Abaqus to analyze the surface stress-strain response. Leveraging symmetry, a quarter-model of a 20-meter pipeline segment (*Φ*1219 mm × 26.4 mm) was constructed. The mesh was refined in the dent region, utilizing C3D8 elements with an approximate size of 10 mm axially and three elements through thickness (Figure 3a). The elastic-plastic behavior of X80 steel was described by a simplified Johnson-Cook model calibrated from tensile tests (Figure 3b). The model was validated against experimental indentation data [23]. As shown in Figure 3c, the simulated load–displacement curve agreed well with the experiment, with maximum relative errors of 3.1% in peak load and 5.2% in residual depth, demonstrating its reliability.

To simulate the in-service loading history, a three-stage simulation procedure was employed (Figure 3d). First, the dent was formed via radially displacing and then retracting the rigid spherical indenter (diameter: 300 mm). Second, the pipeline was subjected to a static internal pressure preload of 12 MPa and subsequently unloaded. Finally, cyclic internal pressure with a triangular waveform (minimum: 1 MPa; variable maximum: *p_max_*) was applied to simulate operational pressure fluctuations.

## 3. Results

### 3.1. Characterization of Pre-Strained Materials

#### 3.1.1. Microstructural Evolution

**Figure 4:**
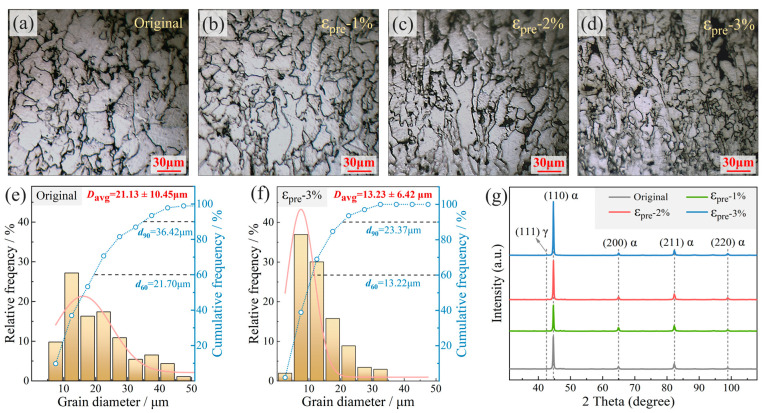
Microstructure (**a**–**d**), grain size statistics (**e**,**f**), and XRD phase analysis (**g**) of the material under different pre-strains.

Figure 4 presents the evolution of microstructure, grain size statistics, and XRD phase analysis of the material subjected to varying levels of pre-strain. The microstructural images in Figure 4a–d reveal a progressive elongation of grains with increasing pre-strain, accompanied by enhanced grain boundary definition. Corresponding grain size distributions, shown in Figure 4e,f, indicate a noticeable reduction in average grain size as pre-strain increases. For instance, after 3% pre-strain, the average grain size decreases by approximately 37.4% compared to the undeformed material, with a narrower size distribution. XRD patterns in Figure 4g demonstrate no significant shift in peak positions or intensity variation across the pre-strain levels, suggesting that the applied pre-strain did not induce detectable lattice strain or phase transformation under the present conditions.

#### 3.1.2. Mechanical Response

**Figure 5:**
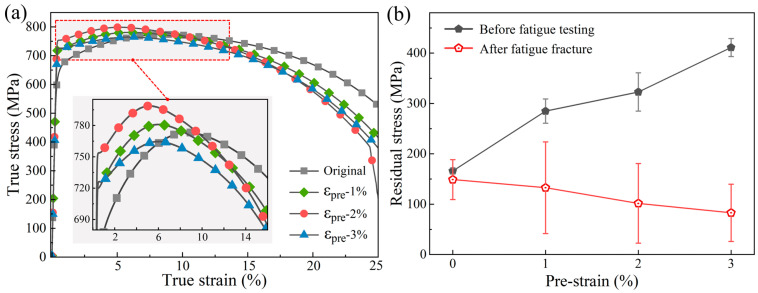
Stress-strain curves of specimens with different pre-strains (**a**) and the corresponding residual stress variations before and after fatigue testing (**b**).

Figure 5 illustrates the mechanical behavior of X80 pipeline steel under various pre-strain conditions. The true stress-true strain curves of the material at various pre-strain levels, as depicted in Figure 5a, were converted from the experimentally measured engineering stress-strain curves using standard relationships [27]. This conversion is necessary because the Johnson-Cook (JC) constitutive model, employed in subsequent numerical analyses, is formulated based on true stress and strain under large deformation conditions. Notably, this conversion is rigorously applicable only within the uniform deformation stage prior to necking. With increasing pre-strain, the flow curves shift upward consistently, indicating pronounced strain hardening. Figure 5b displays residual stress measurements obtained before and after fatigue testing. The results indicate that the initial residual tensile stress increases monotonically with pre-strain, following an approximately linear trend within the investigated range. Following fatigue testing, all specimens exhibited significant relaxation of residual stress. Notably, the extent of relaxation was more pronounced in specimens with higher pre-strain. For instance, the ε_pre_ = 3.0% specimen exhibited the most pronounced stress reduction, from 411 MPa to 83 MPa. Consequently, although specimens with higher pre-strain initially possessed greater residual stresses, this specimen ultimately exhibited the lowest tensile residual stress after fatigue failure.

In addition, Table 3 summarizes the residual plastic strain data for different pre-strain treated specimens. The labels 1.0%, 2.0%, and 3.0% denote the engineering pre-strain levels applied during monotonic tensile loading. The plastic strain values listed correspond to the strain retained after elastic recovery upon unloading, namely the residual plastic strain. The measurements demonstrate good repeatability within each pre-strain group, confirming the reliability of the experimental results. The average residual plastic strain gradually rises with increasing pre-strain, further corroborating the strain-hardening response observed from the stress–strain curves.

### 3.2. Fatigue Behavior of Pre-Strained X80 Steel

#### 3.2.1. Macroscopic Characteristics and Laws of Fatigue Failure

**Figure 6:**
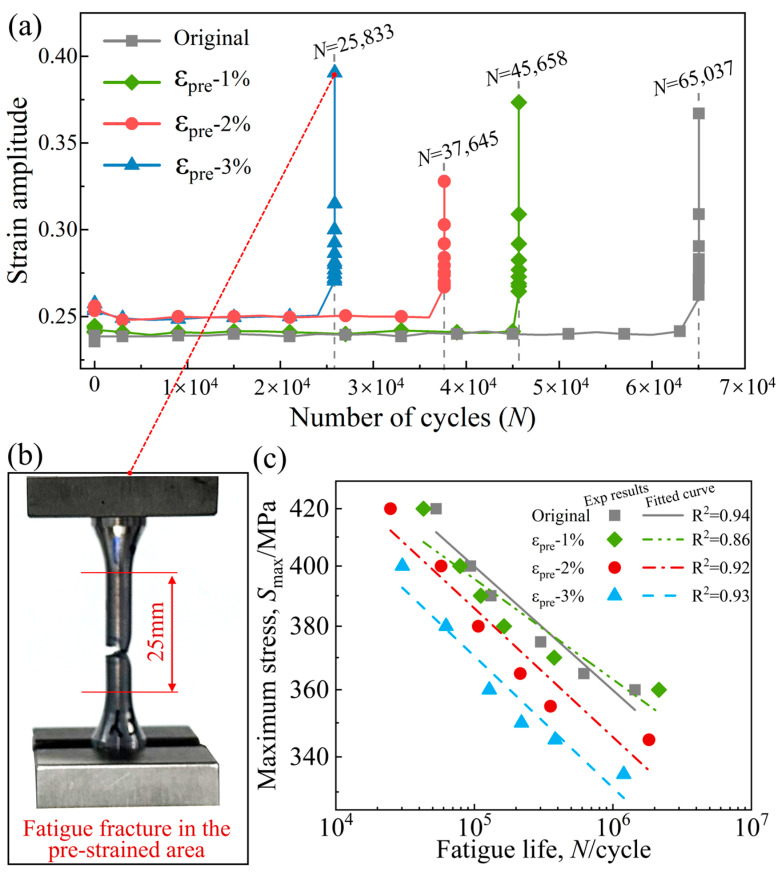
Strain amplitude versus cycles to failure at different pre-strain levels (**a**), with the corresponding fatigue fracture sites (**b**). double-logarithmic *S-N* relationships showing maximum stress (S_max_) versus fatigue life (*N*) with corresponding Basquin-type linear regression fits (**c**).

Figure 6 illustrates the fatigue behavior and macroscopic fracture characteristics of X80 pipeline steel under different pre-strain levels. Figure 6a presents the relationship between strain amplitude and cycles to failure for each pre-strain group. The results demonstrate that as the pre-strain level increases, the fatigue life decreases significantly under the same stress level, suggesting that pre-strain accelerates damage accumulation under cyclic loading. Figure 6b shows a typical fatigue specimen after fracture. The failure occurred within the gauge section, which corresponds to the pre-strained region, and no cracking was observed outside the calibrated length.

The fatigue test results for pre-strained X80 steel specimens are summarized in Table 4. Fatigue life decreases sharply with increasing maximum applied stress. For the non-pre-strained (Original) group, raising the maximum stress amplitude from 360 MPa to 420 MPa resulted in a fatigue life reduction of approximately 1.389 × 10^6^ cycles, corresponding to a 96.3% reduction. Moreover, under identical stress levels, the introduction of pre-strain significantly reduces the material’s fatigue life. At 400 MPa, for instance, compared to the non-pre-strained condition, pre-strains of 1%, 2%, and 3% lead to fatigue life reductions of approximately 15.7%, 38.5%, and 67.7%, respectively. To further elucidate the stress–life relationship, the experimental data from Table 4 were re-plotted in double-logarithmic coordinates (Figure 6c). The resulting *S-N* curves shift systematically downward with increasing pre-strain, confirming its detrimental effect on fatigue performance. One dataset yields a slightly lower coefficient of determination (R^2^ ≈ 0.86), attributed to the deviation from ideal power-law behavior beyond 10^6^ cycles as the material approaches its fatigue limit. Nevertheless, the fitted lines adequately describe the overall trend within the finite-life region.

#### 3.2.2. Fractographic Features Under Different Pre-Strain Levels

**Figure 7:**
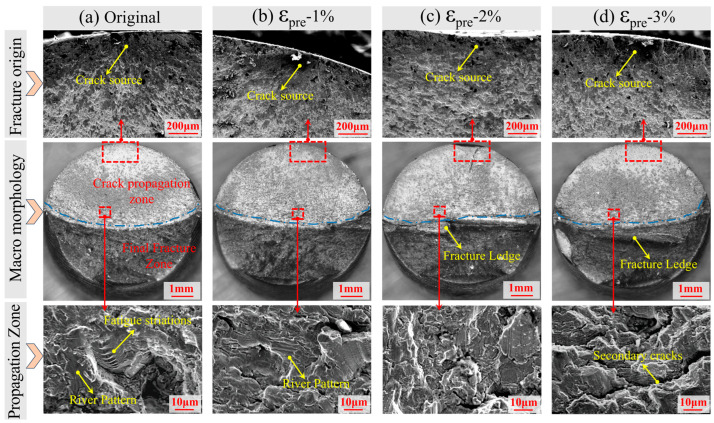
Analysis of macro- and micro-morphology of fatigue fracture surfaces under different pre-strain levels: Original (**a**), 1% pre-strain (**b**), 2% pre-strain (**c**), 3% pre-strain (**d**).

Figure 7 presents the macro- and micro-morphology of fatigue fracture surfaces of X80 steel under different pre-strain levels. Specifically, Figure 7a–d show the fracture morphologies of specimens with 0% (Original), 1% (ε_pre_ = 1.0%), 2% (ε_pre_ = 2.0%), and 3% (ε_pre_ = 3.0%) pre-strain, respectively. All fractures exhibit typical fatigue fracture characteristics, comprising an upper fatigue crack propagation zone and a lower final fracture zone. Cracks in all specimens originated at the specimen surface and propagated inward. As the pre-strain level increases, the number of crack initiation sites increases significantly: the original sample contains only a single crack origin, while the 3% pre-strained specimen exhibits multiple initiation sites on the surface.

At the microscopic scale, distinct fatigue striations are evident in the crack propagation zones of the original and 1% pre-strained specimens, consistent with progressive crack growth under cyclic loading. In contrast, striations become absent in specimens with 2% and 3% pre-strain, where the fracture surface exhibits increased microcracking. This transition suggests that the interaction between pre-stress and cyclic stress alters the crack propagation mechanism; the higher pre-strain promotes the initiation of multiple cracks during fatigue loading, thereby accelerating damage accumulation and the fracture process. In summary, pre-strain not only increases the number and dispersion of crack initiation sites but also modifies the microscopic mode of crack propagation, leading to a significant reduction in fatigue life.

### 3.3. Predicted Fatigue Life of the Dented Pipeline

**Figure 8:**
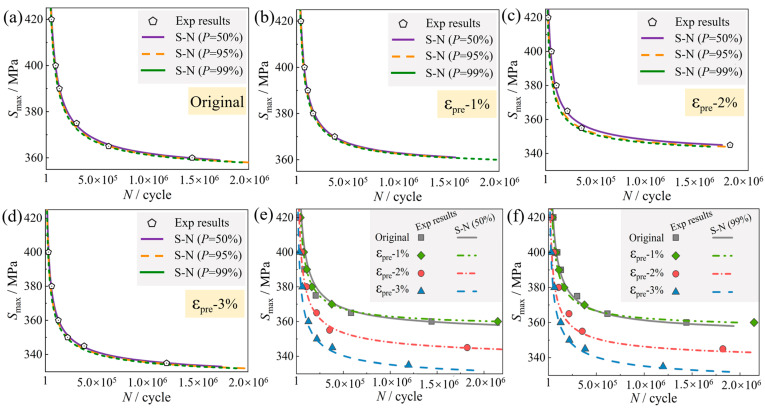
Effect of pre-strain on the *P-S-N* behavior of X80 steel. *P-S-N* curves at various reliability levels for pre-strains of Original, ε_pre_ = 1%, ε_pre_ = 2%, and ε_pre_ = 3% (**a**–**d**). Comparisons of *S-N* curves at *P* = 50% (**e**) and *P* = 99% (**f**), respectively.

Figure 8a–d present the *P-S-N* curves for different pre-strain levels (Original, ε_pre_ = 1%, ε_pre_ = 2%, and ε_pre_ = 3%) at various reliability levels (*P* = 50%, 95%, and 99%), with the corresponding fitting parameters summarized in Table 5. These curves, derived from the optimized single-point likelihood method using standard normal deviates for each reliability level, show good agreement with the experimental data. As expected, for a given stress level, the predicted fatigue life decreases as the required reliability increases, yielding more conservative life estimates. This reduction in life with increasing reliability is influenced by both the pre-strain level and the applied stress.

Figure 8e compares the median *S-N* curves (*P* = 50%). A clear dependence on pre-strain is observed, revealing a dual-stage effect governed by the stress level. At maximum stresses above approximately 367 MPa, fatigue life monotonically decreases with increasing pre-strain. Below this threshold, a non-monotonic trend emerges: life slightly improves from 0% to 1% pre-strain, but then declines at 2% and 3% pre-strain. Consequently, the estimated fatigue limit increases by 1.2% after 1% pre-strain, but decreases by 4.3% and 8.0% after 2% and 3% pre-strain, respectively, compared to the non-pre-strained condition. This indicates that while pre-strain generally degrades high-stress fatigue resistance, a low pre-strain (ε_pre_ = 1%) can marginally enhance low-stress performance, whereas higher pre-strain levels are detrimental across the entire stress range.

### 3.4. Local Mechanical Response in the Dented Pipeline

**Figure 9:**
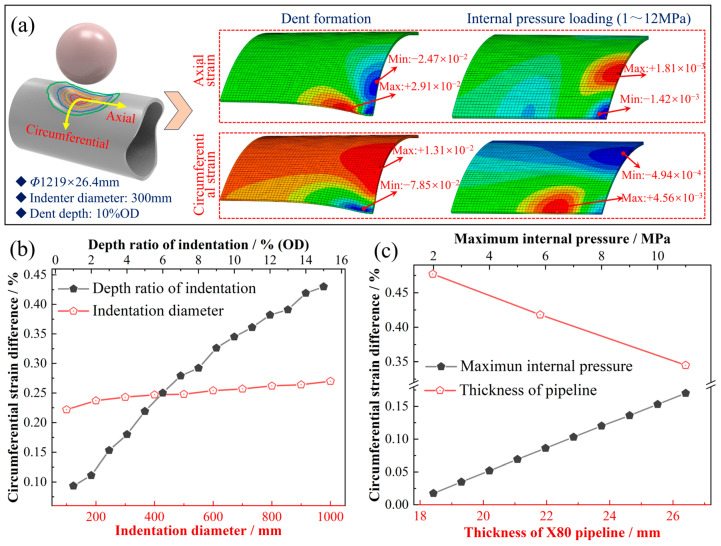
Strain difference distribution characteristics and influencing factors in a pipeline dent region: Contour plots of axial and circumferential strain difference distributions (**a**); Circumferential maximum strain difference as functions of dent dimensions (**b**); Circumferential maximum strain difference as functions of pipeline parameters (**c**).

Crack initiation at the shoulders and subsequent axial propagation characterize the fatigue failure of unconstrained plain dents. As shown in Figure 9, the strain distribution in the dented region of the pipeline and its influencing factors were analyzed based on a finite element model. Figure 9a presents contour plots of the differences in axial and circumferential strain components after the dent stabilizes and during internal pressure fluctuations. Herein, the strain difference is defined as the maximum variation in strain amplitude at each nodal point within the dent-affected zone during one pressure cycle (from 1 MPa to 12 MPa), and is presented as contour plots of the circumferential and axial strain differences. This parameter characterizes the local strain variation under cyclic loading and is extracted from the outer surface nodes of the dent region, where the location exhibiting the maximum strain difference corresponds to the most probable site for fatigue crack initiation. The results indicate that the axial shoulder is consistently subjected to axial tensile strain, while the circumferential shoulder experiences axial compressive strain. Moreover, during the pressure fluctuation cycle, the axial strain amplitude at the circumferential shoulder is more pronounced. In terms of circumferential strain, the axial shoulder remains under circumferential compressive strain, whereas the circumferential shoulder is under circumferential tensile strain. However, during pressure fluctuations, the circumferential strain amplitude at the axial shoulder is greater. Therefore, from the perspective of strain variation in the dent region, both the axial and circumferential shoulders are susceptible to fatigue damage, suggesting that fatigue failure is likely to initiate and propagate along the shoulder edges of the dent.

Additionally, Figure 9b shows the influence of dent size on circumferential strain: with increases in the dent depth ratio and dent radius, the difference in circumferential strain exhibits a linear increasing trend, with depth having a more significant effect. This indicates that dent geometry markedly intensifies the strain concentration effect. Figure 9c further demonstrates that variations in pipeline thickness and internal pressure also significantly affect the circumferential strain distribution. Increasing thickness can mitigate local strain concentration, as evidenced by a decrease in peak strain values with greater thickness. In contrast, raising the internal pressure exacerbates the circumferential strain difference, indicating that the combined action of pressure load and dent geometry leads to more pronounced strain concentration.

## 4. Discussion

### 4.1. Underlying Mechanism of Pre-Strain Effect on Fatigue Damage

#### 4.1.1. Role of Micro-Damage and Dislocation Structures

The experimental results indicate that pre-strain exerts a significant influence on the high-cycle fatigue performance of X80 pipeline steel. As shown in Figure 7, the number of fatigue crack initiation sites increases markedly with higher pre-strain levels. This observation aligns with classical cyclic deformation theories [28,29,30,31], in which plastic pre-strain introduces a substantial density of dislocations, promotes dislocation tangling, and leads to the formation of persistent slip bands (PSBs). These PSBs act as potent stress concentrators and provide preferential sites for crack initiation [32]. Furthermore, pre-strain can induce decohesion of inclusions or second-phase particles from the matrix, resulting in the formation of micro-voids that further facilitate crack nucleation [33]. Under high stress amplitudes (S_max_ > 367 MPa), fatigue life is predominantly governed by the crack initiation and short-crack growth stages. In this regime, the substantial increase in potential crack initiation sites significantly shortens the total fatigue life. Consequently, the detrimental effect of micro-damage becomes dominant, outweighing any potential influence mechanisms, and leads to a progressive degradation in fatigue life with increasing pre-strain.

**Figure 10:**
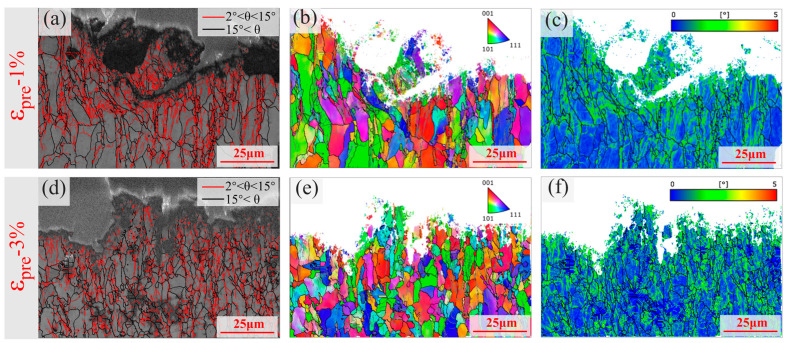
Microstructural characterization via EBSD for specimens with 1% and 3% pre-strain: Grain boundary maps showing grain morphology (**a**,**d**); Inverse pole figure (IPF) maps displaying crystal orientation (**b**,**e**); Kernel average misorientation (KAM) maps reflecting local stress distribution (**c**,**f**).

EBSD analysis provides direct microstructural evidence for the micro-damage mechanisms discussed above. Figure 10 comparatively presents the grain boundary characteristics, grain orientation, and dislocation distribution in specimens with 1% and 3% pre-strain. The grain boundary type maps (Figure 10a,d) reveal a significant increase in the fraction of high-angle grain boundaries (HAGBs) from 36.6% to 45.4% alongside a transition from regular to fragmented grain morphology, indicating that higher pre-strain promotes grain refinement and generates more interfacial defects conducive to crack initiation. Furthermore, the inverse pole figure (IPF) results (Figure 10b,e) show that pronounced grain orientation clustering and a developed deformation texture are present in both the 1% and 3% pre-strained specimens. This microstructural condition promotes micromechanical anisotropy and local stress concentrations during cycling, which in turn accelerates the initiation and early growth of fatigue cracks. Additionally, the kernel average misorientation (KAM) maps (Figure 10c,f) suggest that the 3% pre-strained specimen exhibits significantly higher and more widespread stress concentrations. These high-stress regions correspond to the formation of PSBs and dislocation tangles, thereby acting as preferred sites for fatigue crack nucleation.

In summary, pre-strain beyond the yield point inevitably induces microstructural damage. These microstructural evolutions collectively lead to a significant increase in the number of potential crack initiation sites and an intensification of local stress concentration effects, thereby markedly reducing the fatigue life of the material.

#### 4.1.2. Influence of Residual Stress and Its Cyclic Relaxation

The development of a macroscopic tensile residual stress field (Figure 5b) arises directly from inhomogeneous plastic deformation during pre-straining [28,29,30,31]. As predicted by established mean stress models (e.g., Goodman and Morrow), a tensile mean stress is detrimental to fatigue life because it promotes crack opening and accelerates crack growth [32]. However, the key factor governing fatigue life is not the initial residual stress but its stability under cyclic loading. Post-fatigue residual stress measurements reveal pronounced relaxation (Figure 5b), the extent of which escalates with pre-strain level. This relaxation, indicative of cyclic softening or shake-down, occurs through localized cyclic plastic deformation at stress concentrators, which redistributes and dissipates stored elastic energy [28,29,30,31,32,34].

At low-stress regimes (S_max_ < 367 MPa) with 1% pre-strain. The initial micro-damage is relatively mild. Under cyclic loading, the beneficial relaxation of tensile residual stress becomes dominant. This relaxation consumes part of the cyclic plastic work, effectively reducing the local mean stress and the actual stress amplitude that drives fatigue damage [32]. In this regime, the advantage of a more favorable (lower) mean stress outweighs the detrimental effect of the slight initial damage, resulting in a slight improvement in fatigue life and endurance limit.

For specimens with ε_pre_ = 2% and ε_pre_ = 3% in the low-stress regime. Although residual stress relaxation is even more pronounced (e.g., specimen ε_pre_ = 3%), the initial micro-damage is now severe (Figure 7d). This defect-rich microstructure promotes the formation and coalescence of multiple micro-cracks, ultimately governing the fatigue behavior. Here, the negative impact of irreversible micro-damage dominates over the benefit of stress relaxation, leading to an overall reduction in fatigue performance compared with both the original and the 1% pre-strained samples.

This competitive mechanism elegantly explains the dual effect of pre-strain on fatigue behavior. The findings align with reports for other materials such as SUH660 steel [33], suggesting that the observed interplay represents a more universal material response rather than a case-specific phenomenon.

### 4.2. Correlation Between Material Pre-Strain and Fatigue Life

**Figure 11:**
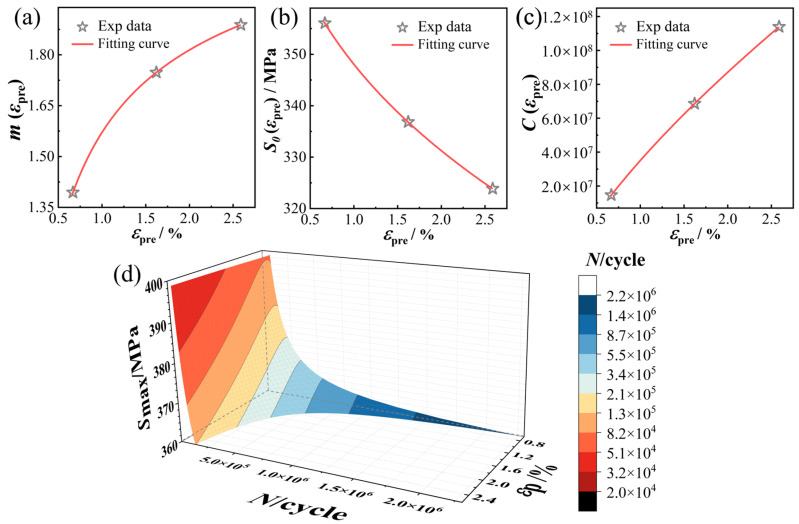
Variation of *S-N* curve parameters $m(\varepsilon_{\mathrm{pre}})$ (**a**), $S_{0}(\varepsilon_{\mathrm{pre}})\;$(**b**), and $C(\varepsilon_{\mathrm{pre}})$ (**c**) with pre-plastic strain; Fatigue life three-dimensional surface of X80 steel as a function of $S_{\max}$ and $\varepsilon_{p}$ (**d**).

The pre-strain described in prior sections encompasses both plastic and elastic components. For fatigue analysis, however, the plastic strain component is often the more relevant variable. This section therefore focuses on establishing quantitative relationships between the pre-plastic strain ($\varepsilon_{p})$ and the parameters governing the *S-N* curve. By combining the measured plastic strain values at different pre-strain levels (Table 3) with the characteristic variations in the *S-N* parameters (Table 5), empirical correlations are proposed for $S_{0}(\varepsilon_{pre})$, $m(\varepsilon_{pre})$, and $C(\varepsilon_{pre})$ in Equation (1) as functions of $\varepsilon_{p}$. The variations in $S_{0}(\varepsilon_{pre})$, $m(\varepsilon_{pre})$, and $C(\varepsilon_{pre})$ with $\varepsilon_{p}$, as described by Equations (3)–(5), are plotted in Figure 11a–c. A clear trend is observed: $S_{0}(\varepsilon_{pre})$ decreases gradually with increasing $\varepsilon_{p}$, whereas $m(\varepsilon_{pre})$ and $C(\varepsilon_{pre})$ show a gradual increase.

(3) $$m(\varepsilon_{\mathrm{pre}})=-0.1427\times {\varepsilon_{p}}^{-0.4162}+2.5413$$

(4) $$S_{0}(\varepsilon_{\mathrm{pre}})=-314.9167\times {\varepsilon_{p}}^{0.3684}+405.7833$$

(5) $$C(\varepsilon_{\mathrm{pre}})=2.1235\times 10^{9}\times {\varepsilon_{p}}^{0.7052}-4.7472\times 10^{7}$$

Substituting Equations (3)–(5) into the *S-N* relationship (Equation (1)) yields a surface function (Equation (6)) that describes the fatigue life of X80 pipeline steel in terms of both pre-plastic strain and applied stress:

(6) $$\begin{array}{ll} N= & (2.1235\times 10^{9}\times {\varepsilon_{p}}^{0.7052}-4.7472\times 10^{7})\times \\ & {\;(S_{\max}+314.9167\times {\varepsilon_{p}}^{0.3684}-405.7833)}^{0.1427\times {\varepsilon_{p}}^{-0.4162}-2.5413} \end{array}$$

By setting $\varepsilon_{p}$ within 0.0066–0.03 and $S_{\max}$ within 360–420 MPa, the resulting fatigue life surface is plotted in Figure 11d. This three-dimensional surface visually captures the combined influence of $\varepsilon_{p}$ and $S_{\max}$ on fatigue life N. For a given stress level, the three-dimensional surface height drops sharply as $\varepsilon_{p}$ increases, reflecting a pronounced reduction in life. Similarly, under a fixed pre-plastic strain, life declines markedly with increasing stress. In practical applications, this formulated surface enables rapid fatigue life estimation for X80 pipeline steel by incorporating specific values of plastic strain and operational stress.

### 4.3. Assessment and Prediction of Fatigue Life for Dented Pipelines

The presence of a dent induces a complex multi-axial stress state on the pipeline surface, which is further intensified under internal pressure, presenting a significant challenge for direct fatigue life calculation. To overcome this limitation, the critical plane approach was employed to reduce the multi-axial fatigue problem to an equivalent uniaxial loading condition, thereby enabling the identification of the principal failure plane. The Smith–Watson–Topper (SWT) parameter [35] was employed to identify the critical plane for multiaxial fatigue assessment. Based on the stress–strain histories obtained from the finite element analysis, the stress and strain tensors at each surface element within the dent region were projected onto candidate material planes via coordinate transformation [36]. An exhaustive angular scanning procedure was implemented in MATLAB R2019b (θ, φ = 0°–180°, 1° increment) to evaluate the SWT value for each orientation. The plane yielding the maximum SWT value was identified as the critical plane. Subsequently, the normal stress history on this critical plane was extracted and corrected for mean stress effects using the Goodman relation [37], while the corresponding normal plastic strain component was obtained through coordinate transformation. SWT-Based Critical Plane Determination for Multiaxial Fatigue Assessment are summarized in the Supporting Information. The fatigue life of each element in the dent region was then determined by substituting the corrected stress amplitude and plastic strain into the experimentally calibrated relationship (Equation (6)).

**Figure 12:**
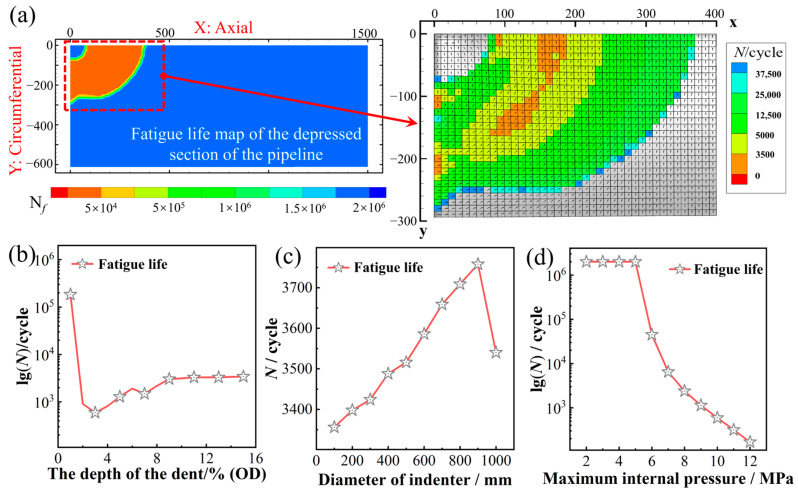
Local fatigue life distribution and critical plane orientations on the dent surface (**a**); Predicted fatigue life of the earliest-failing element in the dent region as a function of: dent depth (**b**), indenter diameter (**c**), and maximum internal pressure fluctuation (**d**).

Analysis of a representative service condition (pipeline: *Φ*1219 × 26.4 mm; indenter diameter: 300 mm; dent depth: 10% OD; p_max_ = 10 MPa) yielded the fatigue life distribution on the unconstrained dent surface, as shown in Figure 12a (with the dent center located at the top-left corner, X-axis: axial direction, Y-axis: circumferential direction). The figure highlights elements with a fatigue life below 5.0 × 10^4^ cycles and indicates the normal directions of their critical planes. These low-life elements are distributed in a ring-like pattern along the dent shoulder, and their critical plane normals exhibit a corresponding annular orientation. The variation in fatigue life with distance from the dent center follows a fluctuating trend of “decrease–increase–decrease–increase” in both the axial and circumferential directions. A pronounced low-life zone is observed around an axial distance of approximately 160 mm. Within this region, the critical plane normals are nearly perpendicular to the pipe axis, suggesting a high likelihood for the initiation of multiple parallel fatigue cracks propagating axially at the shoulder. This prediction aligns with existing experimental observations [38].

Furthermore, based on the finite element simulation results from Section 3.4, the influence of multiple factors (dent depth, indenter diameter, and internal pressure fluctuation) on the fatigue life of the dented area was systematically investigated, as shown in Figure 12b–d. Herein, “fatigue life” is denoted as the predicted number of cycles to failure for the element exhibiting the earliest failure within the dent region, which corresponds to the most critical location for crack initiation according to the SWT model. As illustrated in Figure 12b, with increasing dent depth, the low-cycle fatigue region expands significantly. The minimum life reaches its lowest value (approximately 592 cycles) at a depth of 3% OD. For depths less than 3% OD, the fatigue life is higher due to milder stress concentration, while beyond 9% OD, the life gradually stabilizes. Figure 12c shows that, for a fixed dent depth, the influence of indenter diameter on the minimum surface fatigue life is relatively limited. As the diameter increases, the extent of the low-life zone expands only slightly. The minimum life rises by about 12% when the diameter increases to 900 mm, but exhibits a slight decrease at 1000 mm. This indicates that indenter diameter is not a dominant factor governing fatigue risk. Figure 12d further reveals that the amplitude of internal pressure fluctuation plays a decisive role in the fatigue life at the dent. When the maximum pressure remains below 5 MPa, the predicted fatigue life exceeds 2 × 10^6^ cycles, implying a low risk of failure. With a further increase to *p*_max_ = 9 MPa, the life drops sharply to 1 × 10^3^ cycles. This precipitous drop establishes pressure fluctuation amplitude as the dominant driver of fatigue damage in dented pipelines.

Based on the foregoing analysis, the most vulnerable location for fatigue failure in the dented pipeline region has been identified. Furthermore, parametric analysis prioritized the influencing factors on fatigue life in the following descending order: maximum internal pressure, dent depth, and dent diameter. These findings thereby deliver a guideline for targeted damage prevention in pipeline engineering.

## 5. Conclusions

This study systematically investigated the fatigue behavior and failure mechanisms of dented X80 pipeline subjected to pre-strain damage, integrating material fatigue testing, finite element simulation, and a critical plane-based life assessment methodology. The main conclusions are drawn as follows:

1. Pre-straining beyond the yield point induces microstructural damage, such as grain fragmentation and increased dislocation density. As the pre-strain level increases, the number of potential crack initiation sites rises significantly, and local stress concentration intensifies, leading to a substantial reduction in the material’s fatigue life.
2. A parametric *P-S-N* curve model was established to quantitatively characterize the effect of pre-strain on the fatigue performance of X80 steel. Under low-stress conditions, the slight improvement in the fatigue limit at 1% pre-strain is attributed to beneficial residual stress relaxation. However, at elevated stresses, the accumulation of micro-damage becomes the driving force for degradation, causing fatigue life to significantly decline with higher pre-strain.
3. Based on finite element analysis and the critical plane method, the axial shoulders of unconstrained dents were identified as the most critical regions for fatigue crack initiation. This finding provides a theoretical basis for locating and assessing potential failure sites in dented pipelines.
4. Multi-parameter simulation prioritizes the factors influencing dent fatigue life as: internal pressure fluctuation > dent depth > indenter diameter. This conclusion provides guidance for risk-based ranking and integrity management of dented pipelines.

## Figures and Tables

**Table 1:** Chemical composition of the X80 pipeline steel used for the specimens [22].

Grade	Mn (%)	Si (%)	S (%)	P (%)	C (%)	Fe (%)
X80	1.56	0.53	0.026	0.025	0.20	Balance

**Table 2:** Mechanical property parameters for the X80 pipeline steel.

Young’s Modulus E/GPa	Poisson’s Ratio υ	Yield Strength $\sigma_{y}$/MPa	Tensile Strength $\sigma_{u}$/MPa
197.18	0.3	621	751

**Table 3:** Measured residual plastic strain of X80 steel specimens under different pre-strain conditions (%).

Specimen Group	Test-1	Test-2	Test-3	Test-4	Test-5	Test-6	Average Strain Value
ε_pre_ = 1.0	0.661	0.663	0.662	0.663	0.674	0.682	0.6675
ε_pre_ = 2.0	1.603	1.620	1.618	1.617	1.629	1.639	1.6210
ε_pre_ = 3.0	2.589	2.585	2.580	2.587	2.592	2.597	2.5883

**Table 4:** Fatigue test results for pre-strained specimens (Cycles to failure, *N_f_* ).

Original		ε_pre_ = 1.0%		ε_pre_ = 2.0%		ε_pre_ = 3.0%	
*S*_max_/MPa	*N_f_*	*S*_max_/MPa	*N_f_*	*S*_max_/MPa	*N_f_*	*S*_max_/MPa	*N_f_*
420	52,900	420	42,900	420	24,800	400	30,200
400	93,600	400	78,900	400	57,600	380	62,500
390	131,300	390	111,200	380	106,700	360	128,000
375	300,700	380	163,600	365	214,500	350	218,800
365	616,700	370	376,500	355	354,200	345	382,700
360	1,441,600	360	2,146,800	345	1,821,100	335	1,196,700

**Table 5:** Parameters for *P-S-N* curves of pre-strained fatigue at *P* = 50%, *P* = 95%, and *P* = 99%.

Pre-Strain Level	*P*	*u*_p_	*S*_0_(*ε*_pre_)	*m*(*ε*_pre_)	*m*_p_^’^(*ε*_pre_)	*C*_p_^’^(*ε*_pre_)
Original	50%	0	351.97	1.52	0	3.333 × 10^7^
	95%	−1.645	351.97	1.52	−0.074	1.996 × 10^7^
	99%	−2.326	351.97	1.52	−0.104	1.614 × 10^7^
ε_pre_ = 1%	50%	0	356.04	1.39	0	1.462 × 10^7^
	95%	−1.645	356.04	1.39	0.065	2.017 × 10^7^
	99%	−2.326	356.04	1.39	0.092	2.305 × 10^7^
ε_pre_ = 2%	50%	0	336.82	1.75	0	6.856 × 10^7^
	95%	−1.645	336.82	1.75	0.232	2.155 × 10^8^
	99%	−2.326	336.82	1.75	0.328	3.463 × 10^8^
ε_pre_ = 3%	50%	0	323.85	1.89	0	1.139 × 10^8^
	95%	−1.645	323.85	1.89	0.107	1.918 × 10^8^
	99%	−2.326	323.85	1.89	0.151	2.380 × 10^8^

## Data Availability

The original contributions presented in this study are included in the article/Supplementary Materials. Further inquiries can be directed to the corresponding authors.

## Supplementary Materials

The following supporting information can be downloaded at: https://www.mdpi.com/article/10.3390/ma19050967/s1. References [35,36] are cited in the supplementary materials.
