# Supplementary material for: Effect of Residual Plastic Strain on the Fatigue Failure Mechanism and Service Life Prediction of Dented X80 Pipelines
Source: Materials (Basel). 2026 Mar 3;19(5):967. doi: 10.3390/ma19050967 (PMC12986481; doi:10.3390/ma19050967)
Supplement: Supplementary file 1 [file materials-19-00967-s001.zip › materials-4152748-supplementary.pdf]

Supplementary Material to Section 4.3: SWT-Based Critical Plane Determination for Multiaxial Fatigue Assessment

In Section 4.3, the Smith–Watson–Topper (SWT) parameter was implemented within a critical plane framework to evaluate the multiaxial fatigue damage induced by the combined effects of dent geometry and internal pressure. The adopted procedure is outlined as follows.

First, the multiaxial stress–strain histories obtained from finite element analysis were projected onto potential material planes. The SWT parameter was then evaluated to identify the critical plane, i.e., the orientation that experiences the most severe fatigue damage under cyclic loading. In this work, the SWT parameter serves as a criterion for determining the critical failure plane under multiaxial stress states.

After the critical plane was identified, the corresponding stress and strain components were extracted. The normal stress history on this plane was used to determine the stress amplitude, and a mean stress correction was applied to obtain the equivalent fully reversed stress amplitude. Concurrently, the plastic strain component on the critical plane was obtained through coordinate transformation.

Finally, the corrected stress amplitude, together with the plastic strain on the critical plane, was introduced into the experimentally calibrated plastic strain–fatigue life relationship for pre-strained X80 steel (Eq. (6) in the manuscript), from which the fatigue life was estimated.

The following are the detailed implementation algorithm and steps.

(1) Determination of the Critical Plane

In this study, the multiaxial fatigue damage parameter proposed by Smith et al., namely the Smith–Watson–Topper (SWT) model [S1], was adopted. The SWT parameter is defined as the product of the maximum normal strain range and the maximum normal stress acting on the same material plane:

$$\mathrm{SWT} = \sigma_{\mathrm{n,max}} \left(\frac{\Delta \varepsilon_n}{2} \right) \tag{S1}$$

where $\Delta \varepsilon_n$ denotes the maximum normal strain range on the critical plane, and $\sigma_{\mathrm{n,max}}$ represents the maximum normal stress on that plane.

The determination of the critical plane begins with extracting the stress and strain components at critical locations under different loading conditions. Through coordinate transformation, the stress and strain tensors can be projected onto any arbitrary material plane. The SWT damage parameter is then calculated on each candidate plane, and the plane corresponding to the maximum SWT value is identified as the critical plane. The detailed procedure is summarized as follows [S2].

a) Extraction of Stress–Strain Components

Ased on the finite element results of the dented pipeline, the stress and strain tensor components of elements in the dent region are extracted. The stress tensor can be expressed as:

$$\sigma = \begin{bmatrix} \sigma_{xx} & \tau_{xy} & \tau_{xz} \\ \tau_{xy} & \sigma_{yy} & \tau_{yz} \\ \tau_{xz} & \tau_{yz} & \sigma_{zz} \end{bmatrix} \tag{S2}$$

and the strain tensor as:

$$\varepsilon = \begin{bmatrix} \varepsilon_{xx} & \gamma_{xy} & \gamma_{xz} \\ \gamma_{xy} & \varepsilon_{yy} & \gamma_{yz} \\ \gamma_{xz} & \gamma_{yz} & \varepsilon_{zz} \end{bmatrix} \tag{S3}$$

b) Coordinate Transformation

The stress and strain components were transformed onto arbitrary material planes through coordinate transformation. Let θ denote the angle between the plane normal and the x-axis, and φ denote the angle between the plane normal and the z-axis, as illustrated in Fig-R1.

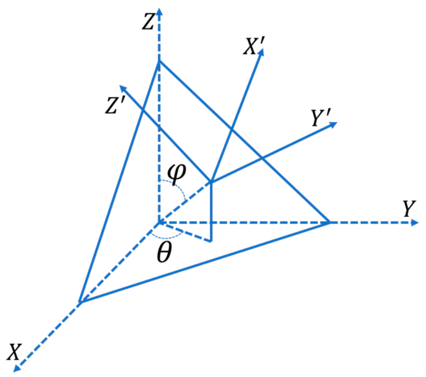

Fig-R1 Coordinate transformation of the material plane

The stress and strain components on an arbitrary plane defined by θ and φ can be obtained using the rotation matrix M:

$$\sigma^{'} = M\sigma M^{T} \tag{S4}$$

$$\varepsilon^{'} = M\varepsilon M^{T} \tag{S5}$$

The rotation matrix M is expressed as:

$$M = \begin{bmatrix} \cos\theta\sin\varphi & \sin\theta\sin\varphi & \cos\varphi \\ -\sin\theta & \cos\theta & 0 \\ -\cos\theta\cos\varphi & -\sin\theta\cos\varphi & \sin\varphi \end{bmatrix} \tag{S6}$$

c) Normal Strain Range on Each Plane

For each candidate plane *i*, the normal strain range is calculated as:

$$\Delta\varepsilon_n^i = \max_{1\le m\le s;m+1\le n\le s}\left\{\left|\varepsilon_{xx}^{'}(m)-\varepsilon_{xx}^{'}(n)\right|\right\} \tag{S7}$$

Where m and n denote the m-th and n-th steps among the s analyzed increment steps, respectively. $\varepsilon^{'}$ represents the strain component after rotation by θ and φ using the rotation matrix, and can be expressed as:

$$\begin{aligned}\varepsilon^{'} = &\cos^2\theta\sin^2\phi\varepsilon_{xx} + 2\sin\theta\cos\theta\sin^2\phi\gamma_{xy} + \\ &2\cos\theta\sin\phi\cos\phi\gamma_{xz} + \sin^2\theta\sin^2\phi\varepsilon_{yy} + \\ &2\sin\theta\sin\phi\cos\phi\gamma_{yz} + \cos^2\phi\varepsilon_{zz}\end{aligned} \tag{S8}$$

d) Maximum Normal Stress on Each Plane

Similarly, the maximum normal stress on plane i is given by:

$$\sigma_{n,max}^i = \max_{1\le m\le s}\left\{\left|\sigma_{xx}^{'}(m)\right|\right\} \tag{S9}$$

Where $\sigma^{'}$ represents the stress component after rotation by θ and φ using the rotation matrix, and can be expressed as:

$$\begin{aligned}\sigma^{'} = &\cos^2\theta\sin^2\phi\sigma_{xx} + 2\sin\theta\cos\theta\sin^2\phi\tau_{xy} + \\ &2\cos\theta\sin\phi\cos\phi\tau_{xz} + \sin^2\theta\sin^2\phi\sigma_{yy} + \\ &2\sin\theta\sin\phi\cos\phi\tau_{yz} + \cos^2\phi\sigma_{zz}\end{aligned} \tag{S10}$$

e) Exhaustive Plane Scanning

In MATLAB, the orientation angles θ and φ were varied from 0° to 180° with an increment of 1°. For each pair (θ, φ), the normal strain range and maximum normal stress were calculated, and the corresponding SWT parameter was evaluated.

The plane corresponding to the maximum SWT value among all candidate planes was identified as the critical plane. The associated orientation angles θ_{max} and ϕ_{max} were recorded for each element.

(2) Fatigue Life Determination of the Dent Surface

After the critical plane was identified, MATLAB was used to process each element individually.

a) Determination of Stress Amplitude

The normal stress history on the critical plane was obtained for each load increment, and the maximum stress S_{max} and minimum stress S_{min} were determined. The stress amplitude on the critical plane was calculated as:

$$S_a = (S_{max} - S_{min})/2 \tag{S11}$$

Since the dented pipeline elements are subjected to asymmetric loading, while the S–N curve

employed in this study was obtained under fully reversed loading (stress ratio R=−1), mean stress correction was required. The Goodman relation was adopted to convert the actual stress state into an equivalent fully reversed stress amplitude:

$$S_{a(R=-1)} = \frac{1}{2}\Delta\sigma \Big/ \left(1 - \frac{\sigma_m}{\sigma_u}\right) \tag{S12}$$

$$\Delta\sigma = S_{max} - S_{min} \tag{S13}$$

$$\sigma_m = (S_{max} + S_{min})/2 \tag{S14}$$

where $S_{a(R=-1)}$ denotes the equivalent fully reversed stress amplitude, $\Delta\sigma$ is the stress range under actual loading, σ_m is the mean stress, and σ_u is the ultimate tensile strength.

b) Determination of Plastic Strain on the Critical Plane

The fatigue life prediction model for pre-strained X80 pipeline steel is given by Eq. (6). To apply this model, the normal plastic strain component on the critical plane must be determined.

The plastic strain components were extracted from the Abaqus simulation results in tensor form:

$$\varepsilon_p = \begin{bmatrix} \varepsilon_{pxx} & \gamma_{pxy} & \gamma_{pxz} \\ \gamma_{pxy} & \varepsilon_{pyy} & \gamma_{pyz} \\ \gamma_{pxz} & \gamma_{pyz} & \varepsilon_{pzz} \end{bmatrix} \tag{S15}$$

Using the previously determined critical plane orientation angles θ_{max} and ϕ_{max}, the plastic strain tensor was transformed via the same rotation matrix:

$$\begin{aligned} \varepsilon_p' = {} & \cos^2\theta_{max}\sin^2\phi_{max}\varepsilon_{pxx} + 2\sin\theta_{max}\cos\theta_{max}\sin^2\phi_{max}\gamma_{pxy} + \\ & 2\cos\theta_{max}\sin\phi_{max}\cos\phi_{max}\gamma_{pxz} + \sin^2\theta_{max}\sin^2\phi_{max}\varepsilon_{pyy} + \\ & 2\sin\theta_{max}\sin\phi_{max}\cos\phi_{max}\gamma_{pyz} + \cos^2\phi_{max}\varepsilon_{pzz} \end{aligned} \tag{S16}$$

The normal plastic strain component on the critical plane, ε_p', was thus obtained.

c) Fatigue Life Calculation

Finally, the corrected stress amplitude $S_{a(R=-1)}$ and the normal plastic strain ε_p' on the critical plane were substituted into the experimentally established fatigue life prediction model for pre-strained X80 steel (Eq. (6) in the manuscript).

The fatigue life of each element in the dent region was thereby determined.

Reference

[S1] Smith, K.N.; Topper, T.; Watson, P. A Stress–Strain Function for the Fatigue of Metals (Stress-Strain Function for Metal Fatigue Including Mean Stress Effect). J Materials 1970, 5,

767–778.

[S2] Hao, C.; Zhu, X.; Wang, W.; Chen, J.; Xu, J.; Lai, J.; Qian, Y.; Wang, P. An Adaptive Multi-Axial Fatigue Model and Fatigue Damage Prediction of the Turnout Rail. *Construction and Building Materials* **2025**, *472*, 140934, doi:10.1016/j.conbuildmat.2025.140934.
